# CAR-巨噬细胞在恶性血液病中的研究进展

**DOI:** 10.3760/cma.j.cn121090-20231103-00248

**Published:** 2024-04

**Authors:** 如冰 郑, 毅 肖

**Affiliations:** 华中科技大学同济医学院附属同济医院血液科，武汉 430030 Department of Hematology, Tongji Hospital, Tongji Medical College, Huazhong University of Science and Technology, Wuhan 430030, China

## Abstract

本文回顾了嵌合抗原受体巨噬细胞（chimeric antigen receptor macrophage, CAR-M）疗法的发展历程，探讨了其在恶性血液系统疾病中的应用进展，介绍了CAR-M相关的临床试验，分析了CAR-M疗法在临床应用中面临的优势与挑战，并对其在未来治疗恶性血液病的应用前景进行了展望。

## Abstract (en)

This article reviews the development history of chimeric antigen receptor macrophage（CAR-M）therapy, discusses its application in malignant hematologic diseases, introduces related clinical trials, analyzes the advantages and challenges faced by CAR-M therapy in clinical application, and looks forward to its future use in the treatment of malignant hematologic diseases.

对于恶性血液病，常用的传统疗法是放射治疗、化学治疗和靶向治疗，而细胞免疫疗法通过对特定的免疫细胞进行修饰，增强免疫细胞本身和机体免疫系统反应对肿瘤细胞的特异性杀伤作用，安全性和有效性都得到了提高。为了克服已经在临床上应用的嵌合抗原受体T细胞（chimeric antigen receptor T-cell, CAR-T）对恶性血液病疗效的不足，研究者提出了将嵌合抗原受体（CAR）引入了其他固有免疫细胞，为恶性血液病免疫治疗提供了革命性的创新。巨噬细胞不仅可以通过细胞表面受体识别肿瘤细胞，直接对其吞噬，而且作为一种免疫抗原提呈细胞可以传递免疫信息增强其他免疫细胞对肿瘤细胞的免疫反应。因此，利用CAR修饰巨噬细胞生成靶向特异性抗原的嵌合抗原受体巨噬细胞（chimeric antigen receptor macrophage, CAR-M）对于恶性血液病具有很大的治疗潜力。

一、CAR-M的概述

1. CAR-M的细胞来源：巨噬细胞是机体固有免疫系统的重要组成成分，在吞噬抗原、分泌细胞因子和呈递抗原中起关键作用，是连接固有免疫和适应性免疫的关键枢纽。巨噬细胞可以在不同的微环境中极化为功能相反的M_1_型和M_2_型[1]。M_1_型巨噬细胞具有高效呈递抗原能力，促炎性和抗肿瘤特性，而M_2_型巨噬细胞主要发挥抑制免疫反应并促进血管生成等作用[2]。巨噬细胞具有高度可塑性，已经分化的M_1_型和M_2_型巨噬细胞也可以相互转化，这两种细胞的平衡在不同血液系统疾病中的作用也不断得到关注。因此，基于巨噬细胞的细胞免疫疗法的核心目标是减少M_2_型抗炎巨噬细胞并增加M_1_型促炎（抗肿瘤）巨噬细胞。

与嵌合抗原受体NK细胞（CAR-NK）类似，在构建CAR-M时使用的巨噬细胞可以从不同来源产生，包括小鼠巨噬细胞系（J774A.1）、人外周血单核细胞（PBMC）和人白血病单核细胞系（THP-1）。PBMC来源的巨噬细胞可以产生大量促炎因子，如IL-8、IL-6和TNF-α[3]，但PBMC来源有限，转染困难，无法有效扩增和冻存。THP-1细胞在用脂多糖（LPS）和干扰素γ（IFN-γ）刺激后可产生M_1_型巨噬细胞，易培养，但用于临床转化上THP-1具有成瘤风险[4]。因此，需要一种新型高效的巨噬细胞来源。最近，Zhang等[5]将PBMC重新编程为诱导多能干细胞（iPSC），产生的异体来源的巨噬细胞经慢病毒转染后成功制备出了表达CAR的巨噬细胞（CAR-iMac），CAR的表达赋予巨噬细胞抗原特异性功能，如促炎细胞因子的分泌，向促炎/抗肿瘤表型极化，增强肿瘤细胞的吞噬能力，以及血液系统恶性肿瘤和实体肿瘤的体内抗肿瘤细胞活性。此项研究技术首次为CAR-M提供了无限来源，具有制备“现货型”产品的潜力。

2. CAR-M的结构设计：CAR-M的构建是将特定的CAR基因转染到巨噬细胞上，使其能够表达CAR，CAR-M通过CAR特异性识别肿瘤细胞相关抗原并与其结合，从而发挥抗肿瘤活性[6]。CAR-M的结构设计与CAR-T和CAR-NK基本相同，主要由三部分融合蛋白组成，包括能够识别特异性肿瘤相关抗原的细胞外结构域、传递信号的跨膜结构域和激活细胞抗肿瘤作用的细胞内结构域。Morrissey等[7]在2018年使用慢病毒载体将Megf10或FcRγ作为细胞内结构域的新型CAR引入J774A.1开发出了第一个CAR-M细胞，最初被称CAR-吞噬细胞。这项研究当时只关注了CAR如何影响吞噬作用而没有进一步探索巨噬细胞其他的抗肿瘤作用，但开启了有关CAR-巨噬细胞免疫疗法研究的新篇章。

目前，第一代CAR-M已经用针对特异性抗原的CAR进行了工程改造，在细胞外结构域上主要常用CD19、CD22、HER2这几种肿瘤相关抗原靶点[8]。第二代CAR-M是在第一代的基础上加入细胞内结构域来完成肿瘤相关抗原的呈递和T细胞活化信号的传递。在第三代CAR-M的设计中采用非病毒载体在体内重编程CAR-M。Kang等[9]使用巨噬细胞靶向聚合物纳米载体将编码CAR和IFN-γ的基因引入巨噬细胞中。IFN-γ基因的加入能将CAR-M从M_2_表型转变为M_1_表型来增强其抗肿瘤效力。

CAR-M目前的研究重点是通过各种细胞内结构域激活和增强吞噬作用。Morrissey等[7]通过筛选一组吞噬受体细胞内结构域，发现来自小鼠吞噬受体库中的Megf10和FcRγ能够作为胞内结构域强烈触发吞噬，将这两种CAR-Ps（CAR-PMegf10和CAR-PFcRγ）转染巨噬细胞作用于CD19^+^淋巴瘤细胞，观察到其吞噬作用显著增强，说明CAR-Ps可以促进巨噬细胞对特定靶点的吞噬作用。之后研究者串联PI3K募集结构域进一步加强了癌细胞吞噬。最后发现表达CAR-Ps的鼠巨噬细胞将共培养中的淋巴瘤细胞数量减少了40%以上。这项研究表明，通过改变CAR结构域可以加强CAR-M的吞噬作用和抗肿瘤活性。这项令人振奋的研究结果可以提供一种思路用于开发更多靶点的CAR-M应用于恶性血液病。

二、CAR-M在恶性血液病中的应用

1. CAR-M与白血病：CAR-M细胞疗法的研究与开发正处于起步阶段，有关恶性血液病的临床前研究和临床研究数量也在逐年增加。CAR-M项目具有良好的前景。

对于恶性血液病中最常见的白血病，为了明确在巨噬细胞表面表达CAR能否促进其对肿瘤的杀伤作用，更有效杀伤白血病细胞。王轶楠等[10]使用THP-1构建了CD19 CAR-M，通过对照试验，检测对人CD19^+^ B淋巴细胞白血病细胞的杀伤作用。结果显示CAR-M对靶细胞的吞噬指数显著增高，并对靶细胞发生了较显著的特异性杀伤。这项研究表明CD19 CAR修饰的巨噬细胞对CD19^+^白血病细胞的吞噬能力较普通的巨噬细胞更强。Zhang等[5]用CAR修饰iPSC分化出的巨噬细胞，成功获得了CD19 CAR-iMac。随后在体外与表达CD19的K562白血病细胞共培养，观察到CAR-iMac抗原依赖性增强的对肿瘤细胞的吞噬活性。还发现CAR介导的信号可诱导CAR-iMac转变为促炎的M_1_亚型。

2. CAR-M与淋巴瘤：通过对CAR结构的设计改造，CAR-M的吞噬能力可以得到加强，同时能够改善肿瘤细胞相关抗原呈递，促进T细胞活化功能。2018年，Morrissey等[7]设计并制备出用于CD19和CD22靶向吞噬作用的CAR并转到巨噬细胞上命名为CAR-Ps。它们的细胞内信号结构域包括除了CD3ζ，还增加了Megf10和FcRγ。结果表明，这些CAR-Ps在体外和体内可以通过特异性抗体介导的相互作用来识别和摄取靶标，对CD19淋巴瘤细胞具有很强的特异性和吞噬能力。Klichinsky的研究团队[11]证明CAR-M在体内可以实现有效的抗肿瘤反应。他们利用嵌合腺病毒载体传递CAR，实现了CAR在人巨噬细胞中针对HER2和间皮素抗原的特异性靶向和持续表达。进一步的体外和体内实验结果证明了这些CAR-M细胞对CD19^＋^血液恶性肿瘤以及HER2和间皮素阳性实体瘤具有更强的吞噬活性和肿瘤杀伤作用。

3. CAR-M与其他恶性血液病：骨髓增生异常综合征（MDS）是起源于造血干细胞的一组异质性髓系克隆性疾病，高风险向急性髓系白血病转化，主要特征是髓系细胞分化及发育异常，表现为无效造血、血细胞减少、造血功能衰竭。最近有研究发现部分MDS患者体内的固有免疫及适应性免疫被异常激活[12]。巨噬细胞作为连接固有免疫和适应性免疫的桥梁，发挥固有免疫的同时也可以将抗原提呈给T细胞以激发Th细胞活化。未来开发CAR-M对于MDS的治疗也具有很大的前景。

CAR-M在恶性血液病中的某些方面还没有得到充分的研究。迫切需要发现新的特异性肿瘤和免疫细胞靶点。CAR-T联合CAR-M免疫疗法在血液系统恶性肿瘤中也值得进一步研究。还需要更多的CAR-M临床试验以评估CAR-M策略的安全性和有效性[13]。

三、CAR-M的临床试验

尽管CAR-M是一种具有广阔前景的肿瘤细胞免疫疗法，但目前为止有关CAR-M的临床试验开展较少，仅启动了3项，且前2项都是针对实体瘤的临床试验[6]。第一项临床试验（NCT04660929）是来自Carisma Therapeutics的CT-0508（HER2靶向CAR-M），来源于嵌合腺病毒载体Ad5f35转导的iPSC[11]，报道了其通过转导THP-1与CD19 CAR-M的临床前开发。该CAR-M通过调节肿瘤微环境（TME）和诱导全身性T细胞应答来减少肿瘤生长并提高生存率，从而显示出良好的治疗能力。此外，CT-0508对HER2肿瘤产生远隔效应，并保护小鼠免受肿瘤复发。对于第二项临床试验（NCT-05007379），FDA授予了其快速通道资格。

最近启动的第3项有关CAR-M的临床试验（NCT05138458）是针对血液肿瘤开展的一项多中心、开放标签、剂量递增和剂量队列扩展的Ⅰ/Ⅱ期临床试验，该试验计划评估MT-101（由来自患者血液的骨髓细胞设计而成）在CD5^+^难治性或复发性T细胞淋巴瘤（R/R-PTCL）患者中的治疗安全性、耐受性和有效性。2022年5月11日，研究团队已对R/R-PTCL患者进行了MT-101的第1次给药。该患者未表现出细胞因子释放综合征（CRS），也无明显的细胞毒性。然而，MT-101的安全性和临床疗效还需要更多的时间来研究，其预计研究完成日期为2024年10月。

四、CAR-M的优势

巨噬细胞是人体吞噬细胞的一种，作为固有免疫细胞，具有免疫信息传递、协同和吞噬处理抗原的功能。与CAR-T细胞疗法相比，CAR-M在临床应用上具有诸多优势。

首先CAR-M可能比其他基于CAR的细胞疗法更安全。外源性CAR-M在体内循环周期有限，可以降低CRS的发生率[14]。其次，使用同种异体的巨噬细胞不会导致GVHD的发生，这对于制备“现货型”CAR-M具有极大的潜力。Zhang等[5]于2020年首次报道了使用表达CAR的iPSC诱导的巨噬细胞（CAR-iMs）进行肿瘤治疗，发现在抗原刺激后，CAR-iMs表现出增强的吞噬作用和M_1_表型偏好。在NSG小鼠血液学和实体瘤模型中，CAR-iMs显示出有效的抗癌活性，且没有明显的不良反应。这项研究提示了CAR-M疗法作为通用疗法的潜力。最后，CAR-M除了对肿瘤细胞具有特异性吞噬作用外，还能促进抗原呈递增强其他免疫细胞介导的肿瘤杀伤作用，实现对高度异质性肿瘤细胞更全面的杀伤作用[15]。

CAR-M在体外和体内已被证明可以低毒性抑制体内肿瘤生长，延长生存期，防止转移，诱导T细胞浸润，并诱导全身抗肿瘤免疫。在新一代CAR-M中，需要细胞因子受体结构域的适应，以进一步提高CAR-M产品的免疫调节和肿瘤杀伤能力[16]。

五、CAR-M的挑战

目前所设计构建的CAR-M疗法作为肿瘤免疫疗法在动物实验中表现较好，但CAR-M的构建和治疗应用仍面临关键挑战，需要克服许多问题才能达到预期的效果。

一是CAR-M制备困难，由于巨噬细胞具有高度可塑和难以扩增的性质，在体外或体内注射后都不会增殖。患者只能接受有限的巨噬细胞，这可能直接导致患者的疗效不佳。二是输入巨噬细胞在体内的迁移特性会严重影响其疗效[17]。注射后，外源性巨噬细胞通过肺部，然后大部分留在肝脏中，不利于肿瘤的治疗，而且还增加了肝损伤的可能性。三是CAR-M细胞转染主要是使用慢病毒和逆转录病毒载体，不仅效率低且存在致癌的风险。最后，由于肿瘤细胞的高度异质性，靶抗原的表达可能不充分。大多数靶向肿瘤细胞相关抗原的免疫细胞可能会向健康组织聚集，出现脱靶毒性[18]。在未来的临床治疗中，应最大限度地提高CAR-M的有效性和安全性，合理选择现有疗法与CAR-M联合使用，可能会对抗肿瘤反应产生协同作用。

在开发肿瘤的细胞免疫疗法时，有研究表明免疫逃逸在肿瘤生长发育中起重要作用，其机制之一是肿瘤细胞表面的CD47通过连接吞噬细胞上表达的信号调节蛋白α（SIRPα）发挥抑制吞噬作用，通过这种方式，CD47可以作为肿瘤细胞“不要吃我”的信号。Ray等[19]发现应用CRISPR / Cas9技术敲除巨噬细胞中的SIRP-α，使CD47无法与其结合，进而可以避免肿瘤细胞发出“不要吃我”的信号，增强巨噬细胞吞噬癌细胞的能力，这为癌症治疗提供了一种新的免疫治疗方法，对未来肿瘤治疗的发展可能具有重要意义。

六、展望

巨噬细胞在机体的固有免疫和适应性免疫中扮演着双重角色，不仅能够吞噬抗原，还具备激发其他免疫反应的能力，通过呈递抗原协同治疗，尤其在恶性血液病的治疗中展现了巨大潜力。CAR-M作为一种创新的细胞免疫治疗策略，为肿瘤治疗开辟了新的广阔天地。基于CAR-T技术的原理设计的CAR-M，着眼于在已有的CAR结构基础上，探索更为高效的CAR分子以激发巨噬细胞的免疫功能。与T细胞和NK细胞相比，巨噬细胞能够极化为具有不同类型和功能的M1/M2型，显示出更为复杂的调节体内平衡的能力，这为开发具有特殊功能的CAR-M技术提供了新的研究方向[20]。随着技术的进步，预期未来CAR-M的应用将超越传统的细胞输注治疗范畴，其在恶性血液病治疗领域中的作用预计将更加重要，为患者带来新的希望。
